# How leadership emotional intelligence promotes team innovation: parallel mediating roles of psychological safety and knowledge sharing

**DOI:** 10.3389/fpsyg.2026.1806655

**Published:** 2026-05-08

**Authors:** Suxuan Xing, Xiaomei Yu, Jun Yu, Jiajia Chen

**Affiliations:** 1 School of Sports Training, Chengdu Sport University, Chengdu, China; 2 School of Physical Education and Health Management, Chongqing University of Education, Chongqing, China; 3 Physical Education Department, Luoxian Middle School, Chenzhou, China

**Keywords:** knowledge sharing, leadership emotional intelligence, parallel mediation effect, psychological security, team innovation

## Abstract

This study examines how leadership emotional intelligence drives team innovation through two distinct mechanisms. Using multilevel structural equation modeling with data from 65 leaders and 391 team members, we reveal that emotionally intelligent leaders influence innovation through both psychological security and knowledge sharing, though with markedly different strengths. Psychological security—the sense that team members can voice ideas without fear—emerges as the primary mechanism, substantially outweighing the effect of knowledge sharing. This dual-pathway mechanism suggests that leadership emotional intelligence operates by first establishing an affective foundation of safety, within which cognitive and behavioral engagement in innovation naturally unfolds. Our findings demonstrate that the relationship between leadership emotional intelligence and team innovation is not straightforward but works through layered mechanisms that vary in their relative importance. This distinction challenges the common assumption that all mediating pathways carry equal weight and highlights why emotionally intelligent leaders are particularly effective: they create the psychological conditions that make team members willing to contribute ideas and take innovative risks. The research contributes to understanding leadership influence on team performance and provides insight into which leadership capabilities matter most for fostering innovation.

## 1 Introduction

The Recommendations of the Central Committee of the Communist Party of China regarding the Formulation of the 14th 5-Year Plan for National Economic and Social Development and the Visionary Goals for the 23rd 5-Year Plan explicitly state that innovation occupies a central role in the context of China's modernization, emphasizing that scientific and technological self-reliance and self-improvement should serve as strategic supports for the nation's development. Data indicate that in 2019, R&D investments in China's innovative enterprises rose by 21.7% year-on-year, and the number of patent applications increased by 14.3%; however, despite these encouraging trends, the efficiency of team innovation remains suboptimal in many organizations, and there is an urgent need to clarify the micro-level (psychological and social) mechanisms that translate organizational R&D inputs into effective team-level innovative outcomes. As the micro-foundation of organizational innovation, team innovation has emerged as a critical driving force enabling organizations to navigate the challenges of the VUCA (volatility, uncertainty, complexity, and ambiguity) era and develop core competitive advantages (Castaneda and Cuellar, 2020). Team innovation refers to the process of generating, developing, and implementing novel and valuable ideas and solutions through knowledge integration and collaboration among team members, guided by shared objectives (Anderson et al., 2014). Team innovation has been examined through various lenses in existing literature, with studies investigating influential factors at multiple levels—from leadership behaviors to team climate and individual member characteristics (Zacher and Rosing, 2015). What stands out in this body of research is the particularly significant role that leadership plays as a contextual factor with profound effects on innovative team outcomes (Chen and Zhou, 2009).

Among leadership competencies, leader emotional intelligence—defined as a leader's ability to identify (detect), understand (interpret), and manage (regulate and use) emotions in oneself and others—merits focused attention for its potential to shape team innovation. Practically, emotionally intelligent leaders (a) detect team members' emotional states and signals early, (b) interpret emotions in relation to task and relational contexts, (c) provide timely emotional support and feedback, (d) model adaptive emotion regulation strategies, and (e) create interactional norms that encourage open expression and constructive dissent. These concrete behaviors help establish a psychological environment conducive to risk-taking, idea exchange, and collaborative problem solving, thereby facilitating team innovation. This relationship can be understood through resource conservation theory, which suggests that when individuals perceive abundant resources initially, they become motivated to invest these resources more fully (Hobfoll et al., 2018). Applied to leadership, emotionally intelligent leaders serve as signals and sources of psychological and social resources (e.g., trust, reassurance, and supportive feedback), which increase team members' felt resource security and thereby motivate greater investment of cognitive and creative effort in innovation. Through this theoretical lens, we can understand how leaders with high emotional intelligence strategically deploy emotional resources in ways that activate innovative potential within teams.

**Figure 1:**
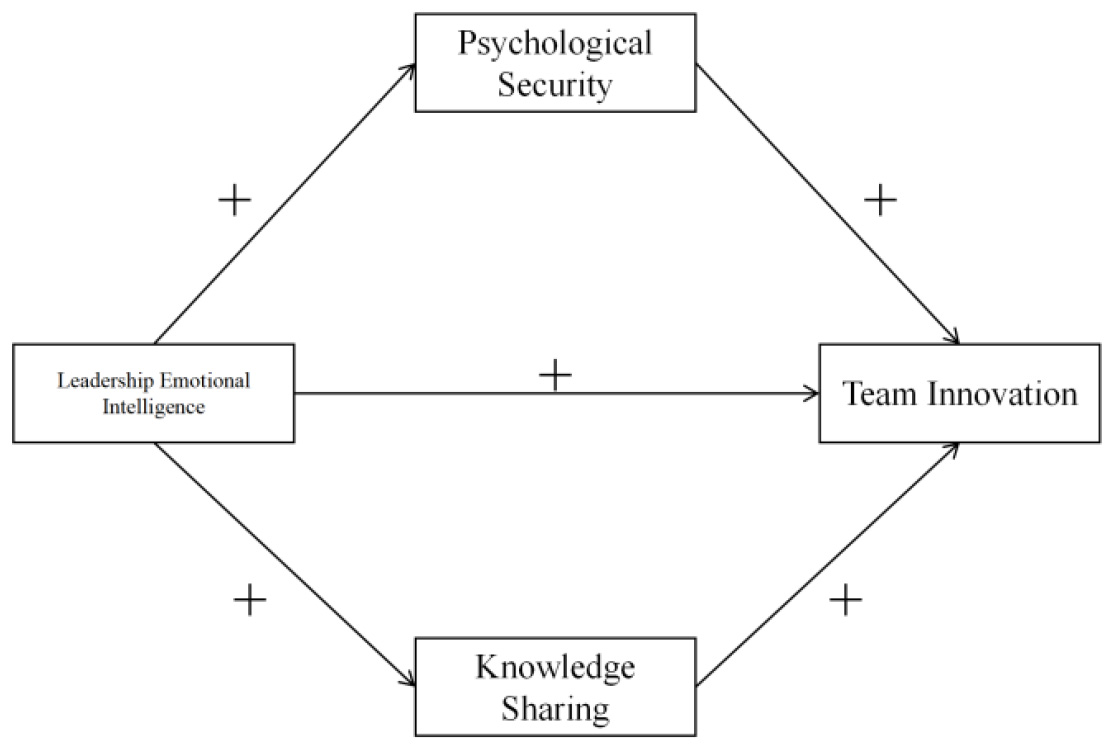
Hypothetical relationship diagram.

Social information processing theory offers another valuable perspective on this relationship. As primary sources of social cues in workplace environments, leaders significantly shape how team members interpret their surroundings (Peng and Wang, 2025). Emotionally intelligent leaders create favorable team psychological conditions, with psychological security emerging as particularly crucial. In such environments, team members feel empowered to voice contrasting viewpoints and engage in open knowledge exchange (Roberts-Olatawura, 2025; Kyambade et al., 2024). The empirical evidence reinforces these theoretical connections. Research has demonstrated that when team members experience psychological security, their innovative behaviors flourish (Ahmed et al., 2025; Zhang and Xu, 2024; Barros de Abreu and Monteiro de Carvalho, 2026). Knowledge sharing appears to serve as a critical connecting mechanism between the psychological security that teams experience and their innovative performance. Exploring these interconnected relationships—how leadership emotional intelligence influences psychological security, which then affects knowledge sharing and ultimately team innovation (Figure 1).

## 2 Literature review and research hypotheses

### 2.1 Leaders' emotional intelligence has a significant effect on team innovation

Leadership emotional intelligence (LEI), conceptualized as a multifaceted competency encompassing the recognition, understanding, utilization, and management of emotions, exerts a substantial influence on team innovation processes. Drawing from Affective Events Theory, leaders' emotional states and expressions elicit emotional responses among team members, consequently modulating their cognitive and behavioral performance (Nimon et al., 2023; Salem et al., 2023). Emotionally intelligent leaders can precisely discern team emotional dynamics, adeptly modulate the team's emotional climate, and strategically provide psychological resources that facilitate innovation within volatile and uncertain environments (Zhou et al., 2020). Emotional contagion theory posits that leaders, leveraging emotional spillover and demonstrative effects, can cultivate a positive team emotional atmosphere that stimulates members' creative ideation and exploratory behaviors (Hoang et al., 2023). From a cognitive resource perspective, leadership emotional intelligence mitigates team members' cognitive load associated with emotional information processing, thereby facilitating increased cognitive engagement in innovation-related tasks (Joseph and Newman, 2010). Moreover, emotionally intelligent leaders adeptly recognize emotional challenges inherent in team innovation processes—including innovation setbacks and interpersonal conflicts—and strategically transmute negative emotional states into innovative motivation through sophisticated emotion regulation mechanisms (Ashkanasy and Dorris, 2017). Empirical investigations consistently demonstrate a significant positive correlation between leadership emotional intelligence and team innovation (Iqbal et al., 2022; Mehmood et al., 2021), with findings substantiated across diverse cultural contexts (Humphrey, 2013; Quan and Guohong, 2013). Multilevel research reveals that leadership emotional intelligence substantially amplifies team innovativeness through strategic interventions: cultivating a conducive emotional climate, augmenting collective emotional competence, and fostering psychological empowerment (Islam and Asad, 2024).

Based on these insights, this study proposes:

Hypothesis 1: Leader emotional intelligence has a significant positive effect on team innovation.

### 2.2 The mediating effect of psychological security between leader emotional intelligence and team innovation

The concept of psychological safety describes a shared belief within teams that interpersonal risks can be taken without fearing negative consequences. In such environments, members feel protected enough to voice genuine opinions, challenge conventional approaches, and openly discuss errors—conditions particularly vital for innovation (Edmondson, 1999). This dynamic becomes especially significant when examining leadership behaviors. Through daily emotional exchanges and response patterns, leaders unconsciously establish behavioral norms that shape team interactions. Those with heightened emotional awareness—the ability to accurately read team members's emotional states and respond with authentic understanding—tend to foster greater organizational transparency and mutual trust (Edmondson and Lei, 2014). When teams develop this psychological safety net, interesting behavioral shifts occur. Members gradually shed defensive postures, engaging more freely in creative experimentation. The typical hesitations around proposing unconventional ideas diminish, replaced by constructive dialogues where concepts undergo rigorous yet respectful evaluation. Research reveals this transformation directly impacts innovation outcomes, with studies documenting measurable improvements in team learning capabilities and cognitive flexibility within psychologically secure groups (Keyan, 2015; Frazier et al., 2017). The connection between leadership approaches and innovation becomes clearer through this lens—particularly for leaders who prioritize emotional intelligence. Their ability to nurture psychological safety appears to function as a key mechanism through which leadership behaviors ultimately translate into innovative team outputs (Newman et al., 2017; Wang, 2018).

Based on these insights, this study proposes:

Hypothesis 2: Psychological security mediates the relationship between leadership emotional intelligence and team innovation.

### 2.3 Mediating effects of knowledge sharing between leader emotional intelligence as well as team innovation

When examining team innovation, knowledge sharing emerges as a crucial process where members exchange insights, translate concepts across professional boundaries, and collectively build new understandings. This complex social activity forms the bedrock of innovative team dynamics. Drawing from resource conservation theory, we can observe how team members become more willing to invest their intellectual assets when they perceive an environment of resource abundance (Hobfoll et al., 2018). The emotional capabilities of team leaders play a particularly interesting role here, as they create psychological conditions that either facilitate or hinder such exchanges (Jamshed and Majeed, 2019). Leaders who read emotional cues effectively tend to navigate the delicate interpersonal dynamics of knowledge sharing with greater skill. They adapt their communication approaches based on team members' emotional states, helping defuse tensions that might otherwise derail productive exchanges (Shariq et al., 2019). This emotional acuity proves especially valuable when addressing the subtle barriers to knowledge sharing that often go unacknowledged in organizational settings—the competitive reluctance to share hard-won insights, the fear of appearing less knowledgeable than peers, or the discomfort of exposing one's mistakes (Akram et al., 2020). Beyond individual interactions, emotionally intelligent leadership shapes how team members collectively manage emotions during collaborative work. This emotional coordination becomes particularly vital when integrating knowledge across disciplinary boundaries where professional languages and priorities may differ dramatically (Jiang and Chen, 2018). As team members bring together their diverse professional perspectives and experiences, they create a richer collective knowledge base that allows for unexpected connections and novel recombinations—the very essence of innovation in complex domains (Gerpott et al., 2020). Empirical research substantiates knowledge sharing's significant positive impact on team innovation (Wang and Wang, 2012), with high-quality knowledge exchange serving as a critical facilitator of team idea generation and implementation (Salehi and Sadeq Alanbari, 2024). Investigations have consistently confirmed knowledge sharing's mediating role between leadership behavior and team innovation (Qiaotian et al., 2020), particularly highlighting leaders' affective competence in enhancing team innovation through knowledge sharing facilitation.

Based on these findings, this study proposes:

Hypothesis 3: Knowledge sharing mediates the relationship between leadership emotional intelligence and team innovation.

## 3 Methods

### 3.1 Objects of study

This research employed a team-level paired survey methodology to collect empirical data, comprising a sample of 65 dyadic leader-team member pairs. The team member sample (*n* = 391) demonstrated a near-equivalent gender distribution (50.13% male, 49.87% female), with a predominantly middle-aged demographic profile (20.2% aged 41–50, 18.41% aged 36–45). The educational composition was characterized by a bachelor's degree majority (53.45%), complemented by a substantial proportion of advanced degrees (24.3%), exemplifying the archetypal contemporary knowledge-intensive team composition. The leadership cohort (*n* = 65) exhibited a predominantly male demographic (64.62%), predominantly clustered within the 31–50 year age bracket (53.85%). The educational profile was predominantly characterized by bachelor's degree holders (50.77%), with professional roles distributed across team leadership (30.77%), departmental management (27.69%), and executive directorates (26.15%). Notably, 29.23% of the sample possessed extensive professional experience exceeding a decade.

### 3.2 Research instrument

#### 3.2.1 Leadership emotional intelligence scale

To measure leadership emotional intelligence, we employed the Wong and Law Emotional Intelligence Scale (WLEIS) (Law et al., 2004). Developed by (Law et al. 2004), this instrument builds upon Mayer and Salovey's theoretical framework (Mayer et al., 2001) and captures four critical aspects of emotional intelligence. The scale contains 16 items distributed across these dimensions: self-emotion assessment (4 items), recognition of others' emotions (4 items), personal emotion regulation (4 items), and practical emotion utilization (4 items). Participants rated each statement on a 7-point Likert scale ranging from “very non-compliant” (1) to “very compliant” (7). Higher total scores reflect greater emotional intelligence capabilities in leadership contexts. When testing the instrument with our research sample, we found strong measurement properties with a Cronbach's alpha of 0.934, demonstrating excellent reliability across all scale components.

#### 3.2.2 Team innovative behavior scale

This research employed the revised Employee Innovation Behavior Scale developed by (Zhang et al. 2016) to evaluate team innovative behaviors. The scale consists of eight items, comprehensively addressing key behavioral manifestations in the innovation process, including problem identification, idea generation, solution support, and implementation verification. Responses are recorded on a 5-point Likert scale (1 = strongly disagree, 5 = strongly agree) to gauge the frequency of employees' willingness to innovate and their corresponding workplace behaviors. The Cronbach's alpha coefficient of the innovative behavior scale was 0.918, indicating very high internal consistency and reliability of the measurement results.

#### 3.2.3 Psychological safety scale

This study employed the Psychological Safety Scale, revised by (Li and Yan 2007), which is based on the original scale developed by (May et al. 2004). The scale assesses team members' perceptions of psychological safety in the workplace through five items, focusing on their concerns regarding negative repercussions while expressing opinions and taking risks. Utilizing a 7-point Likert scale (1 = strongly disagree, 7 = strongly agree), the scale includes four reverse-scored items that synthesize an individual's subjective evaluation of workplace safety. In this study, the psychological safety scale achieved a Cronbach's alpha coefficient of 0.786, reflecting an acceptable level of reliability within social science research, indicating satisfactory measurement stability and consistency.

#### 3.2.4 Knowledge sharing scale

This study employed the revised Knowledge Sharing Scale, as adapted by (Tian 2015) from the original scale developed by (Collins and Smith 2006). The scale comprises seven items rated on a 5-point Likert scale (1 = strongly disagree, 5 = strongly agree) and comprehensively measures two core dimensions of knowledge sharing: willingness to share knowledge (items 1–3) and actual sharing behaviors (items 4–7). Research by (Tian 2015) indicated that the scale exhibits strong reliability, with Cronbach's alpha coefficients of 0.82 and 0.76 for the willingness and behavior sub-scales, respectively. This scale centers on the dynamic process of exchanging and integrating professional knowledge among coworkers within an organization, effectively measuring the degree of knowledge resource flow among team members. In this study, the Cronbach's alpha coefficient for the Knowledge Sharing Scale reached 0.949, confirming exceptional internal consistency reliability and measurement precision.

### 3.3 Research procedures

This study employed a multi-source data collection design, approved by the Chengdu Sport University Ethics Committee (Approval No. 2025-224). The research data have been deposited in the public database Figshare, accessible via doi: 10.6084/m9.figshare.28692056. Data collection occurred from January to March 2024, during which 65 complete teams were recruited through stratified sampling from 16 companies across the manufacturing, technology services, and financial sectors. The research team initially contacted the human resource departments of each company and, upon receiving formal research authorization, utilized an electronic questionnaire system (Questionnaire Star platform) to develop a two-tiered questionnaire. The team leader questionnaire included emotional intelligence scales and demographic information, while the team member questionnaire encompassed scales measuring psychological safety, knowledge sharing, and team innovation. The study employed matching codes for precise leader-member data pairing, enabling each leader to complete the questionnaire and subsequently distribute the matching codes and unique links to their respective team members. The individual in this manuscript has given written informed consent (as outlined in PLOS consent form) to publish these case details.

### 3.4 Data analysis

Raw data were organized in Excel and matched using leadership identification codes, then analyzed using SPSS 26.0. We selected SPSS 26.0 because it accommodates our hierarchical data structure (391 individuals nested within 65 teams) and provides the PROCESS macro (Model 4) for parallel mediation analysis without requiring normality assumptions. Team-level descriptive statistics and Pearson correlations were computed to characterize the variables. The parallel mediation model was tested using bootstrap resampling (5,000 iterations, 95% confidence intervals) to estimate the indirect effects of leadership emotional intelligence on team innovation through psychological safety and knowledge sharing. The sample of 65 teams exceeds power requirements (Cohen's f^2^ = 0.15, power = 0.80), supporting robust estimation.

## 4 Findings

### 4.1 Common method bias test

To mitigate the potential impact of common method bias on the study's results, this research employed a multi-source data collection design supplemented by rigorous statistical testing procedures. Results from Harman's single-factor test indicated that the unrotated first common factor accounted for 32.70% of the variance, significantly below the critical threshold of 50%, thus suggesting no dominance of a single factor in the data structure. Exploratory factor analysis further revealed that all items loaded onto seven distinct factors, consistent with theoretical expectations, yielding a cumulative explained variance of 71.14%, a KMO value of 0.899, and a significant Bartlett's test of sphericity (*p* < 0.001), thus establishing a clear and valid factor structure. Additionally, multiple covariance diagnostics were performed on the relationships among variables; the variance inflation factor (VIF) for all variables remained below 10, with the maximum VIF reaching 5.729, well below the threshold for concern, thereby reinforcing the robustness and credibility of the subsequent analyses.

### 4.2 Descriptive statistics and correlation analysis

To elucidate the fundamental distributional characteristics of the research variables and their inherent correlations, this study conducted descriptive statistics and correlation analyses of the core variables. The data indicated that sample team leaders exhibited a high level of emotional intelligence (M = 5.496, SD = 1.016), a moderate to high level of team innovation behavior (M = 4.005, SD = 0.896), a moderate frequency of knowledge-sharing activities (M = 3.458, SD = 1.201), and a high level of perceived psychological safety (M = 5.259, SD = 1.199). The Pearson correlation analysis revealed significant positive correlations: leadership emotional intelligence with team innovation behavior (r = 0.460, *p* < 0.01), knowledge sharing (r = 0.244, *p* < 0.01), and psychological safety (r = 0.487, *p* < 0.01); psychological safety also correlated significantly with team innovation behavior (r = 0.521, *p* < 0.01); and a significant positive correlation was found between knowledge sharing and team innovation behavior (r = 0.262, *p* < 0.01) (Table 1). These statistical results provisionally support the theoretical hypothesis proposed in this study, suggesting that leadership emotional intelligence may influence team innovation through two parallel pathways: psychological safety and knowledge sharing.

**Table 1:** Pearson correlation—standard format.

	Average value	(statistics) standard deviation	Leadership emotional intelligence	Team innovative behavior	Knowledge sharing	Psychological security
Leadership emotional intelligence	5.496	1.016	1			
Team innovative behavior	4.005	0.896	0.460^**^	1		
Knowledge sharing	3.458	1.201	0.244^**^	0.262^**^	1	
Psychological security	5.259	1.199	0.487^**^	0.521^**^	0.208^**^	1

^*^Indicates statistical significance at the level of *p* < 0.05,

^**^p < 0.01.

### 4.3 Parallel mediation effect test

**Figure 2:**
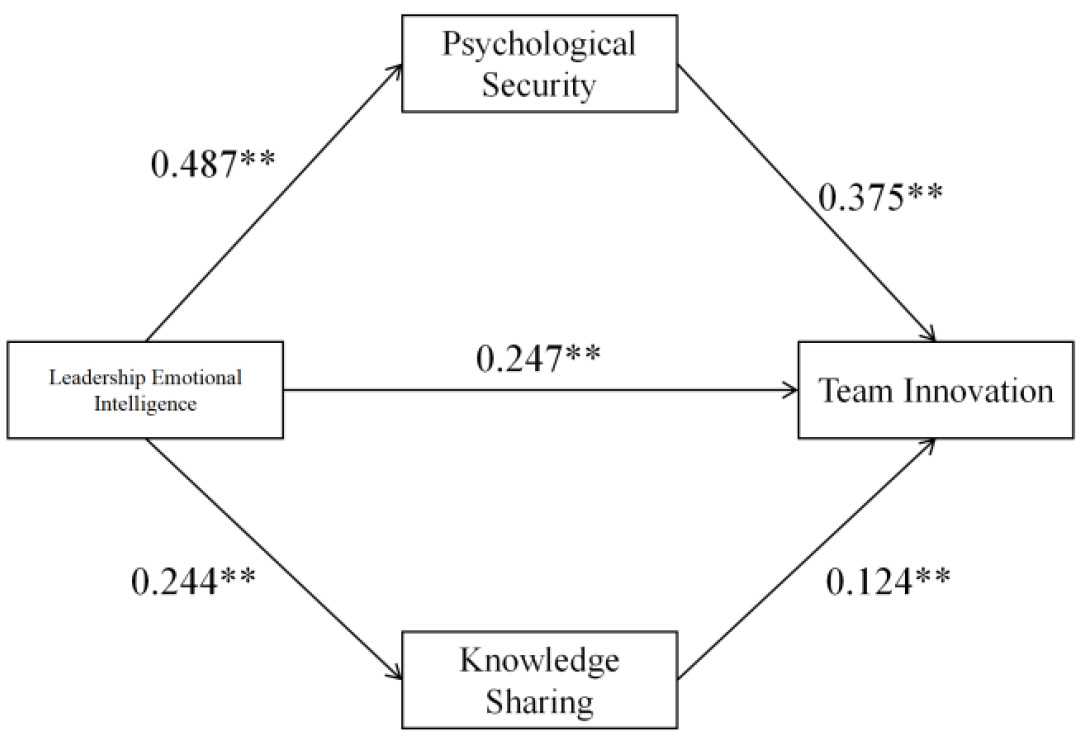
Path diagram of model. **Indicates statistical significance at the level of *p* < 0.01.

Employing Hayes' Process plug-in (Model 4), the study examined the parallel mediating mechanisms of leadership emotional intelligence on team innovation behavior via knowledge sharing and psychological safety, and evaluated the robustness of indirect effects through bias-corrected Bootstrap analysis with 5,000 resamples at a 95% confidence interval. Regression analyses revealed that leadership emotional intelligence significantly predicted both knowledge sharing (β= 0.244, *p* < 0.01) and psychological safety (β = 0.487, *p* < 0.01). Correspondingly, knowledge sharing (β = 0.124, *p* < 0.01) and psychological safety (β= 0.375, *p* < 0.01) emerged as significant predictors of team innovation behavior. Upon controlling for mediating variables, the direct effect of leadership emotional intelligence on team innovation behavior persisted as statistically significant (β= 0.247, *p* < 0.01) (Table 2; Figure 2).

**Table 2:** Path coefficient analysis of the research model.

Outcome variables	Predictor variables	R	R^^2^^	F	β	t
Knowledge sharing	Leadership emotional intelligence	0.244	0.059	24.556	0.244^**^	4.955
Knowledge sharing	Leadership emotional intelligence	0.487	0.237	120.696	0.487^**^	10.986
Team innovative behavior	Leadership emotional intelligence	0.586	0.342	67.076	0.247^**^	5.17
	Knowledge sharing				0.124^**^	2.896
	Psychological security				0.375^**^	7.907

^**^Indicates statistical significance at the level of *p* < 0.01.

The regression models demonstrated progressively increasing explanatory power (R^2^ = 0.059, 0.237, and 0.342, respectively), with all F-tests achieving statistical significance (*p* < 0.001), thereby provisionally substantiating the proposed parallel mediation hypothesis. Subsequent Bootstrap analysis uncovered two significant mediation pathways: (1) leadership emotional intelligence's indirect effect on team innovation behavior through knowledge sharing (β*x*= 0.03, SE = 0.013, 95% CI [0.006, 0.058]), and (2) the indirect effect through psychological safety (β= 0.183, SE = 0.046, 95% CI [0.104, 0.282]). The confidence intervals, excluding zero, substantiated the statistical significance of both mediation mechanisms. The comparative analysis of mediation path effects (C1 = –0.153) revealed a 95% confidence interval of [–0.255, –0.068], which did not encompass zero, thus demonstrating that the psychological safety mediation path exerted a significantly more pronounced mediating effect compared to the knowledge sharing pathway (Table 3).

**Table 3:** Results of parallel mediation effect test.

	Effect	Boot SE	Boot LL CI	Boot UL CI	Relative mediation effect
Aggregate effect	0.46	0.045	0.372	0.549	100.00%
Direct effect	0.247	0.048	0.153	0.342	53.70%
Total indirect effect	0.213	0.047	0.131	0.312	46.30%
Indirect effect1	0.03	0.013	0.006	0.058	6.52%
Indirect effect2	0.183	0.046	0.104	0.282	39.78%
C1	–0.153	0.048	–0.255	–0.068	-

Indirect effect 1: Leadership emotional intelligence → Knowledge sharing → Team innovative behavior.

Indirect effect 2: Leadership emotional intelligence → Psychological security → Team innovative behavior.

C1: Knowledge sharing vs. psychological security.

Comprehensive analysis revealed a total effect of 0.46 for leadership emotional intelligence on team innovation behavior, decomposed as follows: 53.70% direct effect and 46.30% indirect effect. The indirect pathways comprised 6.52% through knowledge sharing and 39.78% through psychological safety. These findings illuminate that leadership emotional intelligence not only directly facilitates team innovation but also indirectly influences innovative processes by: (1) enhancing team psychological safety, and (2) promoting knowledge sharing. Notably, psychological safety emerges as the more pivotal mediating mechanism within this complex influence framework.

## 5 Discussion

Our investigation sought to uncover how a leader's emotional capabilities translate into innovative team outcomes. By testing a parallel mediation model, we found complex relationships between leadership emotional intelligence and team innovation, with psychological safety and knowledge sharing serving as key mediating processes. While both mediators proved significant, the pathway through psychological safety emerged as considerably more influential, highlighting the primacy of emotional dynamics in fostering innovative environments.

Statistical analysis revealed a meaningful connection between leadership emotional intelligence and team innovation, a relationship that makes intuitive sense when viewed through resource conservation theory. Leaders who skillfully manage their emotional landscape appear to create stable psychological conditions where innovation can flourish (Miao et al., 2018). What became apparent in our data was how emotionally intelligent leaders navigate the unpredictable terrain of team interactions-their ability to detect subtle emotional shifts among team members, combined with responsive emotional regulation, builds foundations of trust that encourage creative risk-taking (Zhou et al., 2020). When team members perceive a leader who genuinely understands their emotional experiences, they develop greater psychological safety—a precondition that appears essential for the expression of innovative behaviors (Edmondson, 1999).

This modeling effect subconsciously augments team members' emotional regulation capabilities, generating a positive feedback loop that enables teams to exhibit enhanced resilience amid innovation-related challenges (Extremera et al., 2020). In VUCA environments, the significance of this capability is exponentially heightened, as uncertainty intensifies team emotion management complexities. Leaders with high emotional intelligence can sustain innovation motivation by strategically stabilizing team emotional dynamics and mitigating collective anxiety (Ashkanasy and Dorris, 2017).

Beyond the affective pathway, leadership emotional intelligence indirectly promotes team innovation by facilitating knowledge-sharing behaviors. By creating an open and inclusive emotional climate, leaders encourage team members to share tacit knowledge and creative ideas, thereby stimulating collective intelligence (Nonaka et al., 1996). This mechanism aligns with the parallel mediation path of psychological safety and knowledge sharing, revealing a multilevel action mechanism through which leadership emotional intelligence influences team innovation. Domestic scholarly investigations (Quan and Guohong, 2013) further emphasize the centrality of emotional intelligence in innovative team dynamics, reinforcing the generalizability of these research findings.

The findings demonstrated that psychological safety significantly mediated the relationship between leadership emotional intelligence and team innovation, accounting for 39.78% of the total effect and illuminating a critical psychological mechanism underlying emotional intelligence's innovative influence. Extant literature converges on the understanding that team psychological safety serves as a pivotal antecedent variable propelling team innovation (Newman et al., 2017), with leaders' emotional competence emerging as a primary mechanism for cultivating this psychological state (Edmondson and Lei, 2014). Theoretically, psychological safety, conceptualized as a collective belief system, effectively mitigates the interpersonal risks encountered by team members when articulating innovative concepts (Edmondson, 1999). It is precisely this capacity for risk mitigation that enables emotionally intelligent leaders to cultivate supportive environments where team members feel empowered to explore innovative trajectories (Klem and Schlechter, 2008).

As (Artinger et al. 2025) elucidate, psychological safety functions not merely as a protective buffer but as an essential prerequisite for innovative engagement, particularly in processes characterized by concurrent uncertainty and risk. Leaders with sophisticated emotional intelligence demonstrate an exceptional capacity to discern team members' emotional requirements, offering precise and timely emotional support that systematically attenuates innovation-related anxiety (Mayer et al., 2016), and (Li and Li 2024) further illuminated how leaders' emotional expression patterns critically influence the team's psychological climate, thereby establishing a foundational mechanism for augmenting team psychological safety.

The observed mediating mechanism finds additional support in social cognitive theory, which posits that leadership emotional intelligence modulates team members' environmental safety perceptions through demonstrative modeling and emotional transmission processes (Yang et al., 2012; Gu, 2016). Emotionally regulated and inclusive leadership cultivates an environment wherein team members experience reduced apprehension when articulating novel ideas, and the substantial proportion of the total effect attributable to psychological safety unequivocally suggests that the fundamental mechanism translating leadership emotional intelligence into team innovative outcomes resides in its affective pathway (Xiao et al., 2023). Consequently, this research offers nuanced organizational guidance for enhancing leadership emotional competence and cultivating psychologically supportive environments conducive to team innovative potential.

Turning to the cognitive-behavioral dimension, the research uncovered a significant yet comparatively modest mediating effect of knowledge sharing between leadership emotional intelligence and team innovation, accounting for 6.52% of the total effect. This finding illuminates how leadership emotional intelligence catalyzes knowledge dissemination and ultimately influences team innovative dynamics. Extant literature substantiates knowledge sharing as a fundamental catalyst for team innovation (Hu and Randel, 2014), with leaders' emotional intelligence emerging as a pivotal mechanism in the social exchange processes governing knowledge flow (Wang et al., 2014). From a knowledge management perspective, leaders with sophisticated emotional intelligence can. cultivate a conducive knowledge sharing environment, systematically mitigate knowledge impediments (Malik, 2022), and attenuate psychological barriers by precisely discerning and addressing team members' emotional requirements while reinforcing interpersonal emotional connectivity.

Social exchange theory further illuminates how leaders catalyze employees' innovative performance through strategic delegation and empowerment, with emotional intelligence serving as a critical moderating mechanism that amplifies the strategic value of tacit knowledge exchange (Hou et al., 2021). Empirical investigations substantiate these theoretical propositions: (Shariq et al. 2019) demonstrated that key opinion leaders exert a direct and positive influence on knowledge sharing, underscoring the strategic imperative for organizations to cultivate learning-oriented employees' participation in knowledge exchange initiatives, while- (Shafique et al. 2023) elaborated that knowledge-oriented leadership effectively facilitates both tacit and explicit knowledge dissemination, with emotional intelligence functioning as a critical moderating mechanism.

Notably, (Jamshed et al. 2017) revealed a significant positive correlation between leadership emotional intelligence and knowledge sharing, demonstrating knowledge sharing's substantial mediating role in the relationship between emotional intelligence and team effectiveness-an observation that aligns with (Othman and Abdullah 2012) which posit that leadership emotional intelligence can strategically attenuate negative emotional dynamics during knowledge sharing, thereby facilitating innovative processes. The comparatively modest mediating effect of knowledge sharing, when juxtaposed with the affective pathway, suggests that the cognitive-behavioral mechanism exerts a more limited yet nonetheless meaningful influence in the relationship between leadership emotional intelligence and team innovation. This finding resonates with (Flinchbaugh et al. 2020), who observed that affective factors constitute a more foundational and sustained mechanism in team innovative dynamics. In conclusion, leadership emotional intelligence augments team innovation through knowledge sharing mechanisms, offering organizations strategic insights for enhancing leadership emotional competence and optimizing knowledge management practices.

## 6 Implications, limitations, and future directions

### 6.1 Theoretical implications

The parallel mediation model reveals that affective mechanisms substantially outweigh cognitive-behavioral pathways in translating leadership emotional intelligence into team innovation, with psychological safety accounting for 39.78% of the total effect compared to 6.52% for knowledge sharing. This empirical finding recalibrates existing theoretical understanding by demonstrating that psychological safety operates as the foundational mechanism through which leader emotional competencies catalyze team-level creative engagement. The dominance of the affective pathway extends resource conservation theory by positioning emotionally intelligent leaders as primary sources of psychological resources that enable teams to sustain the vulnerability inherent in innovative endeavors. Concurrently, the model clarifies why knowledge management and knowledge-sharing initiatives, though significant, achieve limited impact without the prerequisite affective climate—a distinction that has remained theoretically underspecified in prior research. These findings suggest that future theoretical frameworks examining leadership influence on team outcomes should afford greater theoretical weight to emotional climate variables as primary mechanisms rather than peripheral moderators, thereby reshaping how scholars conceptualize the pathways connecting leader attributes to collective performance.

### 6.2 Practical implications

For organizational implementation, the findings argue for a reorientation of leadership development priorities toward emotional intelligence capabilities that directly construct psychological safety—including emotion perception, contextual emotional interpretation, and modeling emotionally supportive behaviors that signal receptiveness to novel ideas and learning from failure. The substantial mediation effect of psychological safety indicates that organizations can generate meaningful innovation gains by investing in leader development programs targeting these specific emotional competencies rather than generic leadership training. Concurrently, the findings do not diminish the importance of knowledge management infrastructure; rather, they suggest that knowledge-sharing platforms and incentive structures function most effectively when embedded within psychologically safe team environments that have been established through emotionally intelligent leadership. This implies a sequenced organizational strategy: prioritize leader emotional intelligence development and team psychological safety cultivation as foundational work, then deploy knowledge management mechanisms that operate upon this affective foundation. Such an approach recognizes that organizational innovation capacity depends not on any single intervention but on the coordinated deployment of affective, cognitive, and structural enablers in appropriate sequence.

The cross-sectional design employed in this research prevents the establishment of causal directionality and obscures the temporal dynamics through which leadership emotional intelligence, psychological safety, knowledge sharing, and team innovation co-develop and mutually reinforce one another. Additionally, despite the multi-source data collection approach, the sample encompassed 65 teams distributed unevenly across industries, which may constrain the generalizability of findings to specific sectors or organizational contexts. The parallel mediation specification, while clarifying the relative potency of distinct mechanisms, did not examine potential sequential dependencies between psychological safety and knowledge sharing—a gap that leaves open the question of whether affective conditions must necessarily precede cognitive engagement for innovations to emerge. Longitudinal research designs would enable observation of causal development patterns and permit investigation of how the proposed mechanisms unfold dynamically across organizational time horizons. Future studies might explore the chain-mediation pathway of leadership emotional intelligence → psychological safety → knowledge sharing → team innovation, thereby illuminating whether sequential effects exist and whether the affective-to-cognitive transition represents a necessary progression. Introducing moderating variables such as team diversity, task complexity, and organizational culture would further delineate the boundary conditions under which affective mechanisms dominate vs. contexts where cognitive pathways assume greater salience. Cross-cultural examination would also clarify whether the prominence of psychological safety mechanisms generalizes across cultural contexts or whether emotional expression norms and safety constructs operate distinctly in non-Western organizational settings. These extensions would strengthen the theoretical framework and provide more nuanced guidance for organizations operating across diverse institutional environments.

## 7 Conclusions and recommendations

This study developed and validated a theoretical model illustrating how leadership emotional intelligence influences team innovation through the parallel mediating mechanisms of psychological safety and knowledge sharing, elucidating both the process and its relative significance. The findings indicate that leadership emotional intelligence not only directly fosters team innovation but also indirectly enhances it through increased psychological safety and knowledge sharing. Notably, the mediating effect of the affective pathway (psychological safety) is significantly stronger than that of the cognitive pathway (knowledge sharing). These findings enrich the theoretical framework concerning the relationship between leaders' emotional competence and team innovation, underscoring the centrality of emotional factors in facilitating team creative performance. Based on these findings, it is recommended that organizations enhance leaders' capacity to identify, understand, and manage emotions, establish psychologically safe team environments, and implement effective knowledge sharing mechanisms to systematically improve team innovation performance, thus supporting sustainable competitive advantage in uncertain environments.

## Data Availability

The datasets presented in this study can be found in online repositories. The names of the repository/repositories and accession number(s) can be found below: https://figshare.com/s/a89b52ba544a198f4406.
