# Supplementary material for: How leadership emotional intelligence promotes team innovation: parallel mediating roles of psychological safety and knowledge sharing
Source: Front Psychol. 2026 May 8;17:1806655. doi: 10.3389/fpsyg.2026.1806655 (PMC13194025; doi:10.3389/fpsyg.2026.1806655)
Supplement: Supplementary file 1 [file Table_1.docx]

# LEADER QUESTIONNAIRE

**Dear Leader,**

Thank you for participating in this academic research on "Leadership Emotional Intelligence and Team Innovation." This study aims to investigate how leadership emotional intelligence influences team innovation through psychological safety and knowledge sharing.

This questionnaire is conducted anonymously. All data will be used solely for academic research, and we will maintain strict confidentiality of all information you provide. The questionnaire takes approximately 10 minutes to complete.

**[IMPORTANT NOTE] To match team data, please carefully create your matching code and remember it to share with team members later.**

**Q1: Please select your company/organization [Single Choice] ***

- ☐ Alibaba Group (A)
- ☐ Baidu (B)
- ☐ China Construction Bank (C)
- ☐ China Eastern Airlines (D)
- ☐ Evergrande Group (E)
- ☐ Fosun International (F)
- ☐ State Grid Corporation of China (G)
- ☐ Huawei Technologies (H)
- ☐ IKEA China (I)
- ☐ JD.com (J)
- ☐ iFlyTek (K)
- ☐ Lenovo Group (L)
- ☐ Midea Group (M)
- ☐ CATL (N)
- ☐ L'Oréal China (O)
- ☐ Shanghai Pudong Development Bank (P)

**Q2: Please enter the name of your department/team [Fill in the blank] ***

- ☐ __________________________________________________ (Hint: Please enter the formal name of your department/team)

**Q3: Please create your leader identity matching code [Fill in the blank] ***

- ☐ ______________________________ (Format: L-Company Code-First Letters of Name-Last Four Digits of Phone)

**Q4: Gender [Single Choice] ***

- ☐ Male
- ☐ Female

**Q5: Age [Single Choice] ***

- ☐ Under 30
- ☐ 31-40
- ☐ 41-50
- ☐ Over 51

**Q6: Educational Level [Single Choice] ***

- ☐ Associate Degree or Below
- ☐ Bachelor's Degree
- ☐ Master's Degree
- ☐ Doctorate or Above

**Q7: Years in Current Position [Single Choice] ***

- ☐ Less than 1 year
- ☐ 1-3 years
- ☐ 4-6 years
- ☐ 7-10 years
- ☐ More than 10 years

**Q8: Size of Team You Manage (Direct Reports) [Single Choice] ***

- ☐ 5 or fewer
- ☐ 6-10
- ☐ 11-20
- ☐ 21 or more

**Q9: Leadership Emotional Intelligence Scale [Matrix Scale] ***

Hint: Based on your actual situation, please rate the following statements:

Scoring Standard: 1=Strongly Disagree, 2=Disagree, 3=Somewhat Disagree, 4=Neutral, 5=Somewhat Agree, 6=Agree, 7=Strongly Agree

**[Self-Emotion Assessment]**

| **Item** | **1** | **2** | **3** | **4** | **5** | **6** | **7** |
| --- | --- | --- | --- | --- | --- | --- | --- |
| I usually know the reasons for my certain feelings |  |  |  |  |  |  |  |
| I understand my own emotions very well |  |  |  |  |  |  |  |
| I can truly understand my feelings |  |  |  |  |  |  |  |
| I often know why I feel happy or unhappy |  |  |  |  |  |  |  |

**[Emotion Management Ability]**

| **Item** | **1** | **2** | **3** | **4** | **5** | **6** | **7** |
| --- | --- | --- | --- | --- | --- | --- | --- |
| When facing difficulties, I can control my temper |  |  |  |  |  |  |  |
| I am very able to control my emotions |  |  |  |  |  |  |  |
| When I'm angry, I can usually calm down in a very short time |  |  |  |  |  |  |  |
| I have very strong control over my emotions |  |  |  |  |  |  |  |

**[Self-Motivation Ability]**

| **Item** | **1** | **2** | **3** | **4** | **5** | **6** | **7** |
| --- | --- | --- | --- | --- | --- | --- | --- |
| I can usually set goals for myself and strive to achieve them |  |  |  |  |  |  |  |
| I often tell myself that I am a capable person |  |  |  |  |  |  |  |
| I am someone who can encourage myself |  |  |  |  |  |  |  |
| I often encourage myself to do my best |  |  |  |  |  |  |  |

**[Recognition of Others' Emotions]**

| **Item** | **1** | **2** | **3** | **4** | **5** | **6** | **7** |
| --- | --- | --- | --- | --- | --- | --- | --- |
| I can usually guess my friends' emotions from their behavior |  |  |  |  |  |  |  |
| My ability to observe others' emotions is very strong |  |  |  |  |  |  |  |
| I can acutely perceive others' feelings and emotions |  |  |  |  |  |  |  |
| I understand the emotions of people around me very well |  |  |  |  |  |  |  |

**Thank you for completing this questionnaire!**

**[IMPORTANT NOTES]**

1. Please remember your leader identity matching code: [Refer to Q3 Answer]

2. Please send the team member questionnaire link along with your matching code to your team members

3. Team Member Questionnaire Link: <https://www.wjx.cn/vm/Y8p3MXU.aspx#>
