# Supplementary material for: How leadership emotional intelligence promotes team innovation: parallel mediating roles of psychological safety and knowledge sharing
Source: Front Psychol. 2026 May 8;17:1806655. doi: 10.3389/fpsyg.2026.1806655 (PMC13194025; doi:10.3389/fpsyg.2026.1806655)
Supplement: Supplementary file 2 [file Table_2.DOCX]

# TEAM MEMBER QUESTIONNAIRE

**Dear Team Member,**

Thank you for participating in this academic research on "Leadership Emotional Intelligence and Team Innovation." This study aims to investigate how leadership emotional intelligence influences team innovation through psychological safety and knowledge sharing.

This questionnaire is conducted anonymously. All data will be used solely for academic research, and we will maintain strict confidentiality of all information you provide. The questionnaire takes approximately 15 minutes to complete.

**[IMPORTANT NOTE] Please ensure you have obtained your direct supervisor's "Leader Matching Code," as this information is crucial to this research.**

**Q1: Please select your company/organization [Single Choice] ***

- ☐ Alibaba Group (A)
- ☐ Baidu (B)
- ☐ China Construction Bank (C)
- ☐ China Eastern Airlines (D)
- ☐ Evergrande Group (E)
- ☐ Fosun International (F)
- ☐ State Grid Corporation of China (G)
- ☐ Huawei Technologies (H)
- ☐ IKEA China (I)
- ☐ JD.com (J)
- ☐ iFlyTek (K)
- ☐ Lenovo Group (L)
- ☐ Midea Group (M)
- ☐ CATL (N)
- ☐ L'Oréal China (O)
- ☐ Shanghai Pudong Development Bank (P)

**Q2: Please enter the name of your department/team [Fill in the blank] ***

- ☐ __________________________________________________ (Keep it consistent with what your leader entered)

**Q3: Please enter your direct supervisor's/team leader's matching code [Fill in the blank] ***

- ☐ ________________________________________ (Format: L-Company Code-First Letters-Last Four Digits)

**Q4: Please create your member identity matching code [Fill in the blank] ***

- ☐ ___________________________________ (Format: M-Company Code-First Letters-Last Four Digits)

**Q5: Gender [Single Choice] ***

- ☐ Male
- ☐ Female

**Q6: Age [Single Choice] ***

- ☐ 25 or younger
- ☐ 26-35
- ☐ 36-45
- ☐ 46 or older

**Q7: Educational Level [Single Choice] ***

- ☐ Associate Degree or Below
- ☐ Bachelor's Degree
- ☐ Master's Degree
- ☐ Doctorate or Above

**Q8: Years in Current Team [Single Choice] ***

- ☐ Less than 1 year
- ☐ 1-3 years
- ☐ 4-6 years
- ☐ 7 years or more

**Q9: Time Working with Current Direct Supervisor [Single Choice] ***

- ☐ 6 months or less
- ☐ 6 months to 1 year
- ☐ 1-3 years
- ☐ 3 years or more

**Q10: Psychological Safety [Matrix Scale] ***

Hint: Based on your actual feelings in your current work environment, please rate how much you agree with the following statements:

Scoring Standard: 1=Strongly Disagree, 2=Disagree, 3=Somewhat Disagree, 4=Uncertain, 5=Somewhat Agree, 6=Agree, 7=Strongly Agree

| **Item** | **1** | **2** | **3** | **4** | **5** | **6** | **7** |
| --- | --- | --- | --- | --- | --- | --- | --- |
| In my work, I don't need to always be cautious and careful |  |  |  |  |  |  |  |
| In my work environment, there are always people behind me causing trouble, making my work efforts in vain |  |  |  |  |  |  |  |
| My work environment is full of various potential threats |  |  |  |  |  |  |  |
| Once you make a small mistake at work, the consequences will be very severe |  |  |  |  |  |  |  |
| In the work environment, there are always people constantly finding fault with me |  |  |  |  |  |  |  |

**Q11: Knowledge Sharing [Matrix Scale] ***

Hint: Based on your actual behavior in your team, please rate how much you agree with the following statements:

Scoring Standard: 1=Strongly Disagree, 2=Disagree, 3=Neutral, 4=Agree, 5=Strongly Agree

| **Item** | **1** | **2** | **3** | **4** | **5** |
| --- | --- | --- | --- | --- | --- |
| To keep up with the company's new concepts, new products, or service levels, I am willing to share new ideas and knowledge with colleagues |  |  |  |  |  |
| I am willing to share my professional knowledge to help new projects or ideas proposed by the company be effectively implemented |  |  |  |  |  |
| I am willing to share knowledge with colleagues |  |  |  |  |  |
| By sharing knowledge among colleagues, it helps us improve each other's knowledge level |  |  |  |  |  |
| By exchanging and combining knowledge with colleagues, I can complete work tasks more quickly than working independently |  |  |  |  |  |
| When encountering work problems, we can skillfully solve them through exchanging and combining knowledge |  |  |  |  |  |
| When work tasks are completed, I find that sharing knowledge with colleagues allows me to learn many new things from them |  |  |  |  |  |

**Q12: Team Innovation Behavior [Matrix Scale] ***

Hint: Based on your actual behavior in your team, please rate how much you agree with the following statements:

Scoring Standard: 1=Strongly Disagree, 2=Disagree, 3=Uncertain, 4=Agree, 5=Strongly Agree

| **Item** | **1** | **2** | **3** | **4** | **5** |
| --- | --- | --- | --- | --- | --- |
| I often look for opportunities to improve work methods and work processes |  |  |  |  |  |
| I often try to adopt new methods to solve problems that arise in my work |  |  |  |  |  |
| I often think about problems from different angles |  |  |  |  |  |
| I don't miss any opportunity to understand and discover problems |  |  |  |  |  |
| I often suggest implementing new work methods in the company |  |  |  |  |  |
| I often take risks to support new ideas or creativity |  |  |  |  |  |
| I often introduce new work methods to my colleagues |  |  |  |  |  |
| I often verify the effectiveness of new work methods |  |  |  |  |  |

**Thank you very much for completing this questionnaire survey!**
